# Supplementary material for: Peptidomic Characterization and Amino Acid Availability after Intake of Casein vs. a Casein Hydrolysate in a Pig Model
Source: Nutrients. 2023 Feb 21;15(5):1065. doi: 10.3390/nu15051065 (PMC10005447; doi:10.3390/nu15051065)
Supplement: Supplementary file 1 [file nutrients-15-01065-s001.zip › nutrients-2209596-supplementary.pdf]

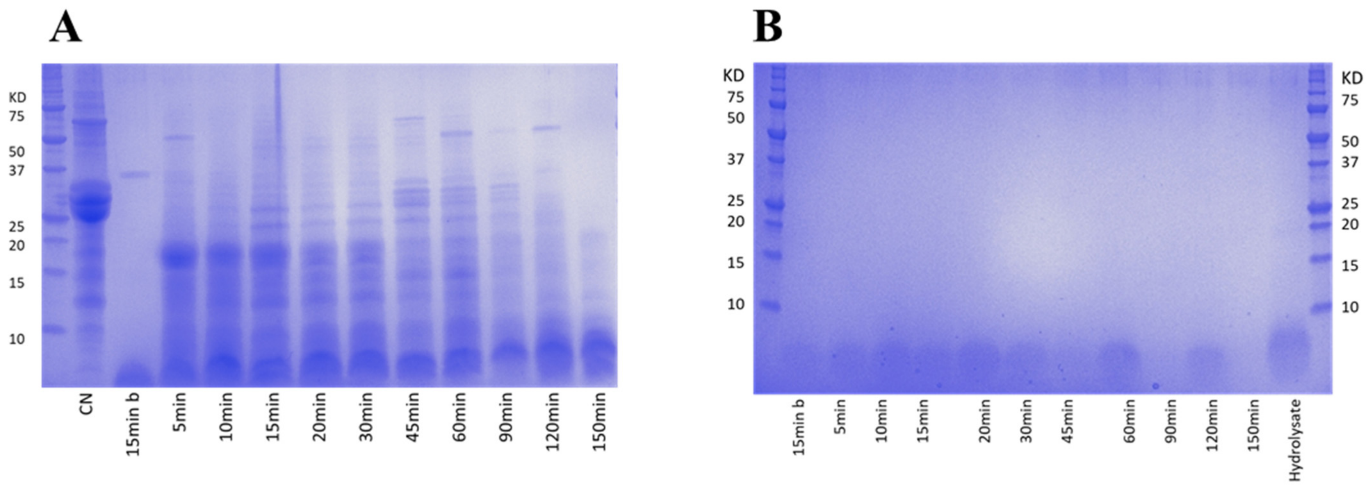

Figure S1. Electrophoretic profile of pig duodenal effluents at different times of digestion after intake of casein (A) and hydrolysate (B) compared with the undigested substrate.

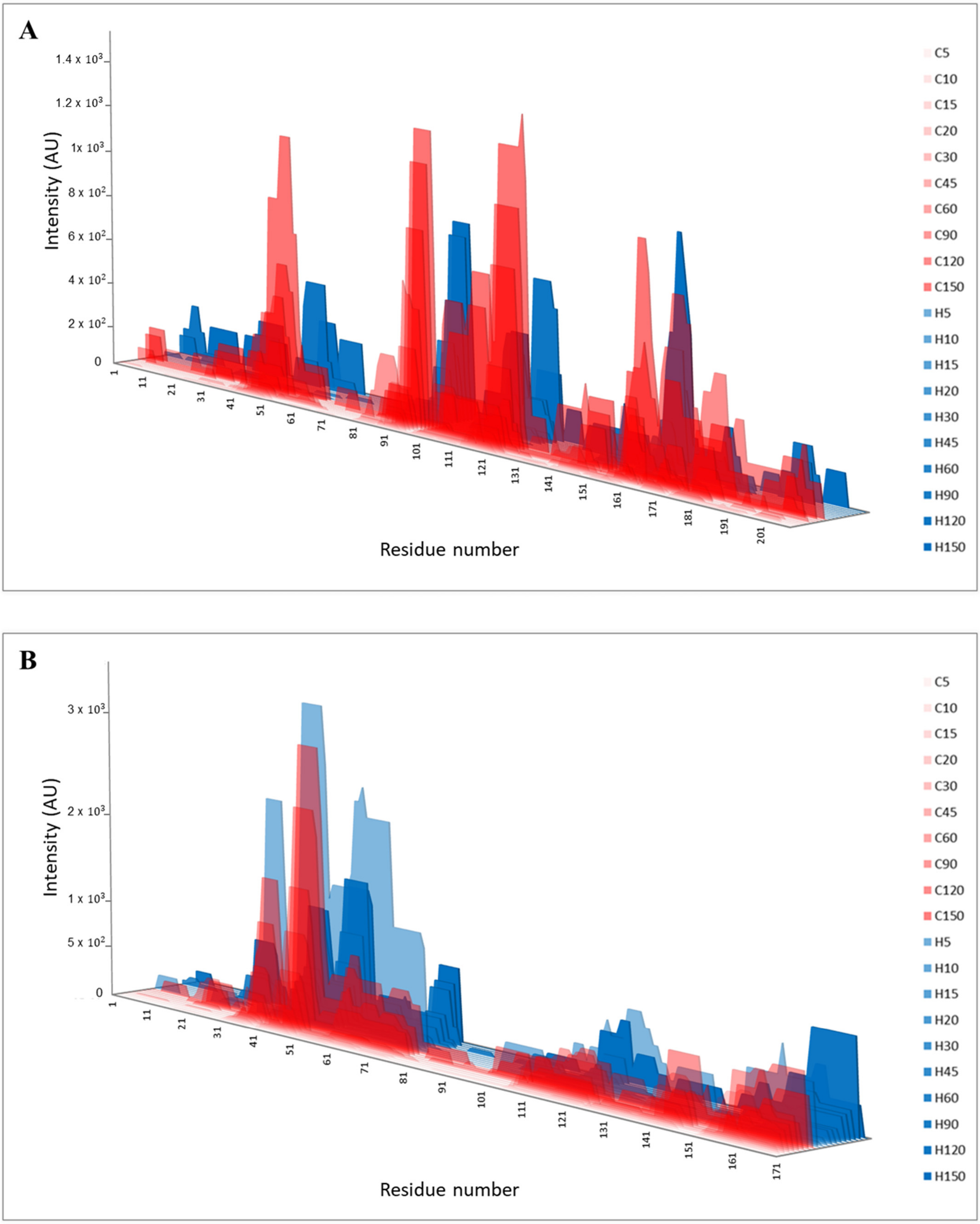

Figure S2. Identified peptides in pig duodenal digests after intake of casein (C) or hydrolysate (H) over collection time represented by area graphs. The height (Y-axis) is proportional to the sum of the identified peptide intensities. Profile peptides from α_{s1}-casein (A) and κ-casein (B).

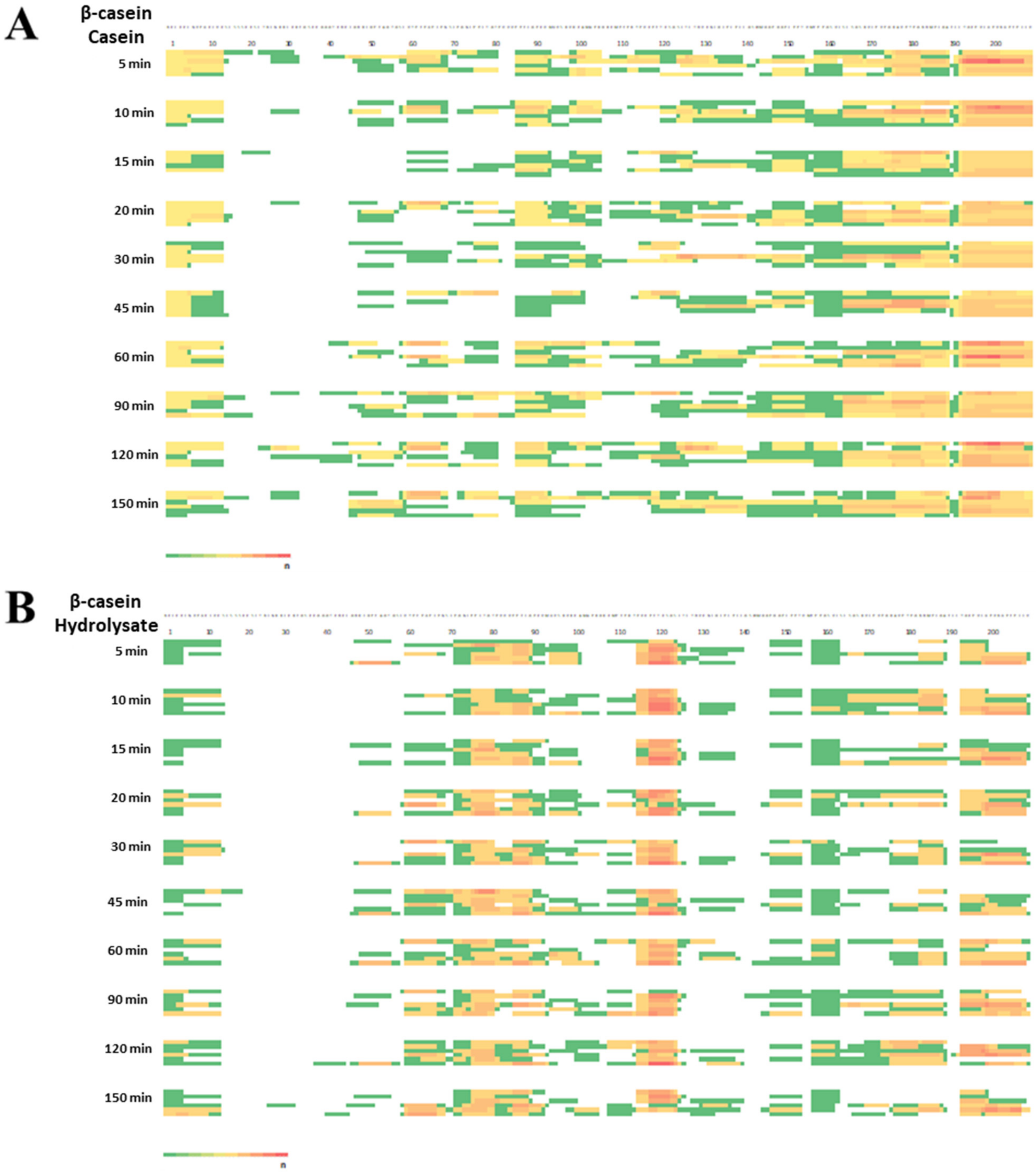
A
β-casein
Casein
5 min
10 min
15 min
20 min
30 min
45 min
60 min
90 min
120 min
150 min
B
β-casein
Hydrolysate
5 min
10 min
15 min
20 min
30 min
45 min
60 min
90 min
120 min
150 min

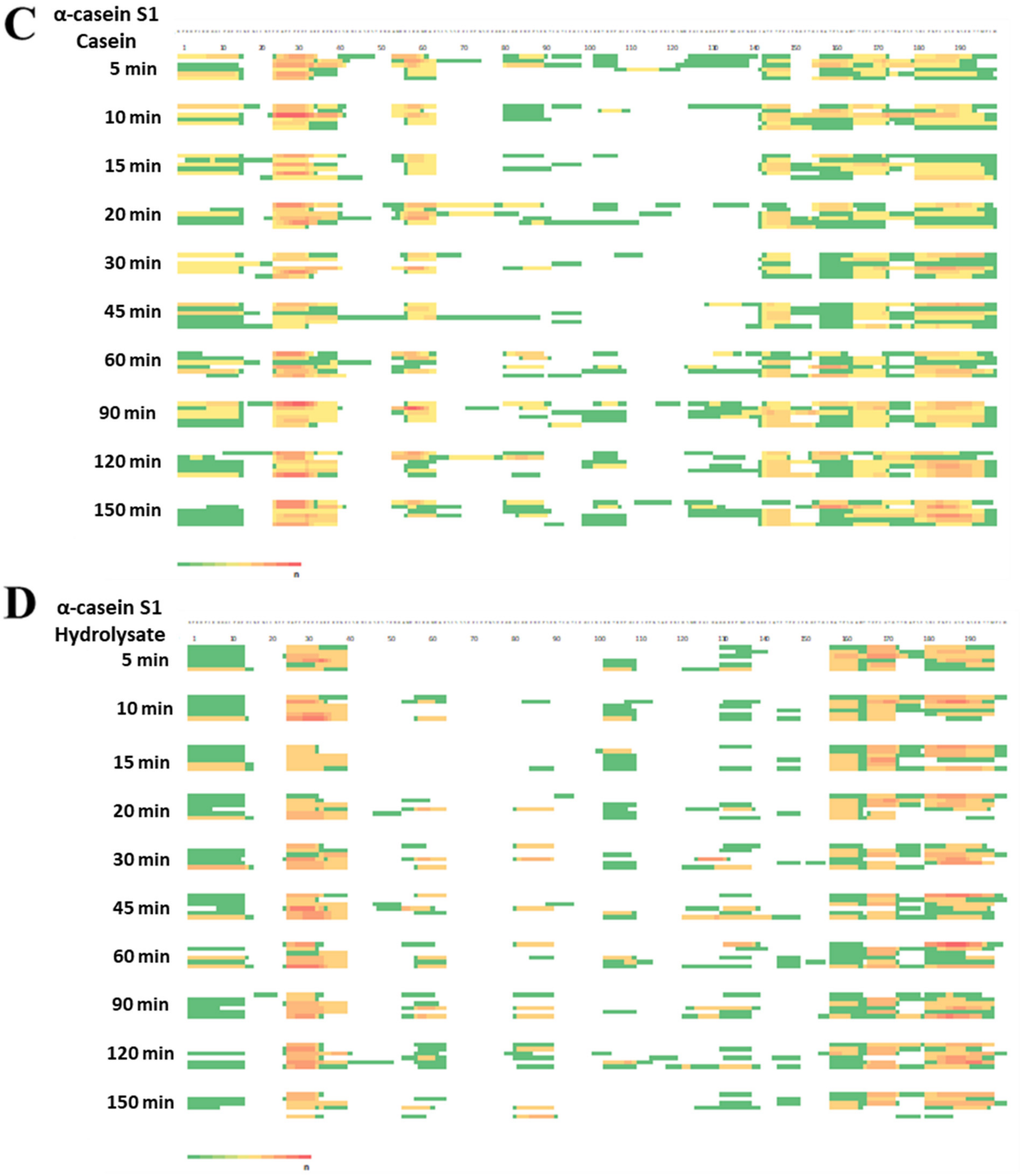

Figure S3. Alignment of peptides identified in pig duodenal effluents (*n* =6) collected at different times of digestion after intake of casein (A, C) or hydrolysate (B, D) and represented as heatmaps of β-casein (A, B) and α_{s1}-casein (C, D). Each line at each time point represents a different subject (*n* =6).

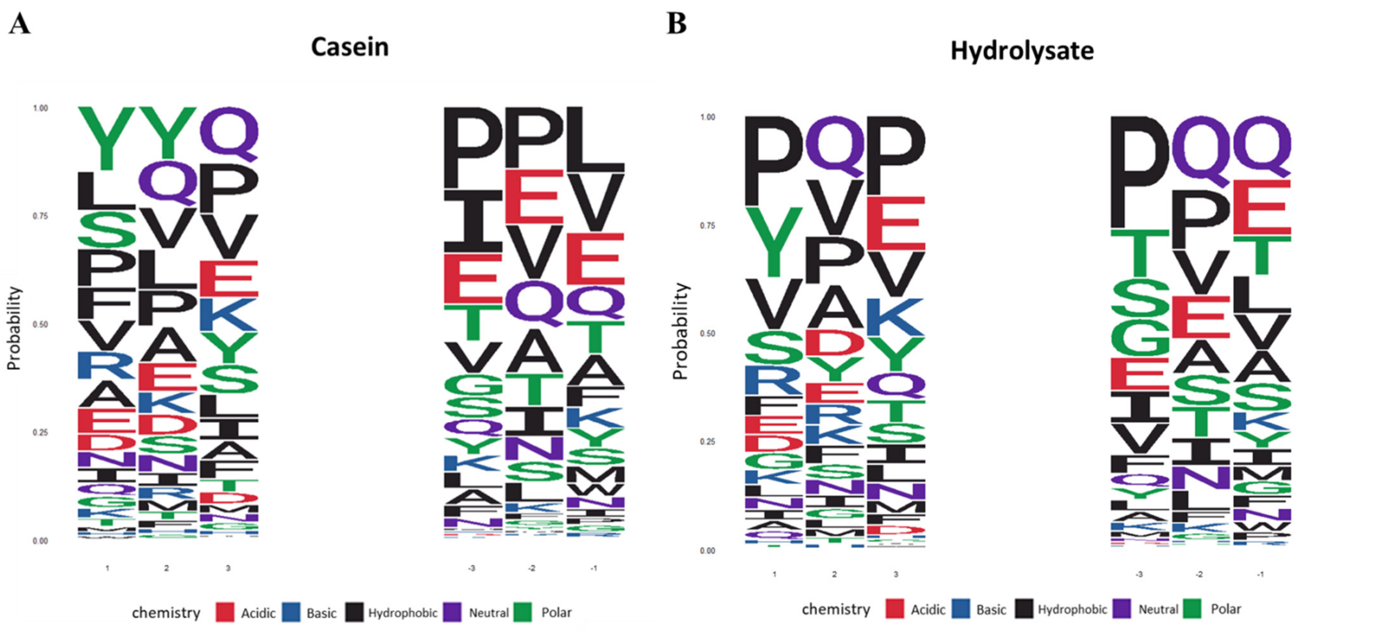

Figure S4. SeqLogo analysis of terminal amino acid residues corresponding to the peptides identified in pig duodenal effluents (*n* =6) at basal (-15 min), 5, 10, 15, 20, 30, 45, 60, 90, 120, and 150 min after intake of casein (A) or hydrolysate (B).

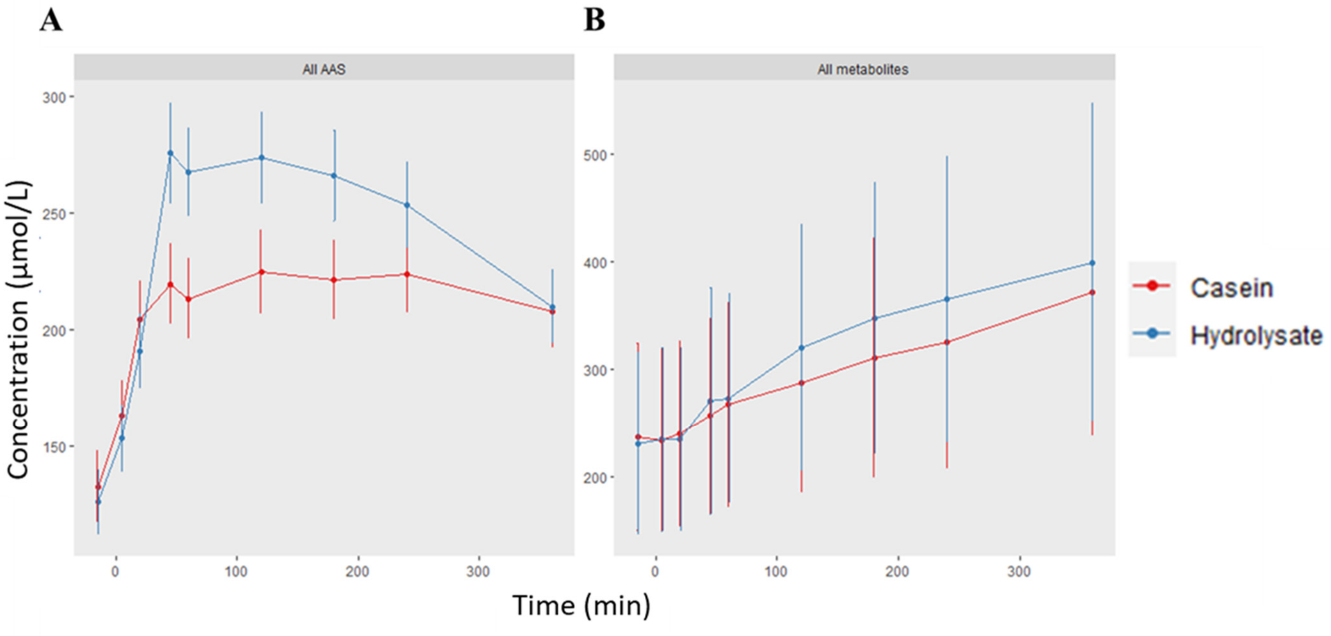

Figure S5. Total amino acids (A) and metabolites (B) determined in plasma at baseline (-15 min) and 5, 20, 45, 60, 120, 180, 240 and 360 min after intake of casein or hydrolysate in the pig model (*n* =6).

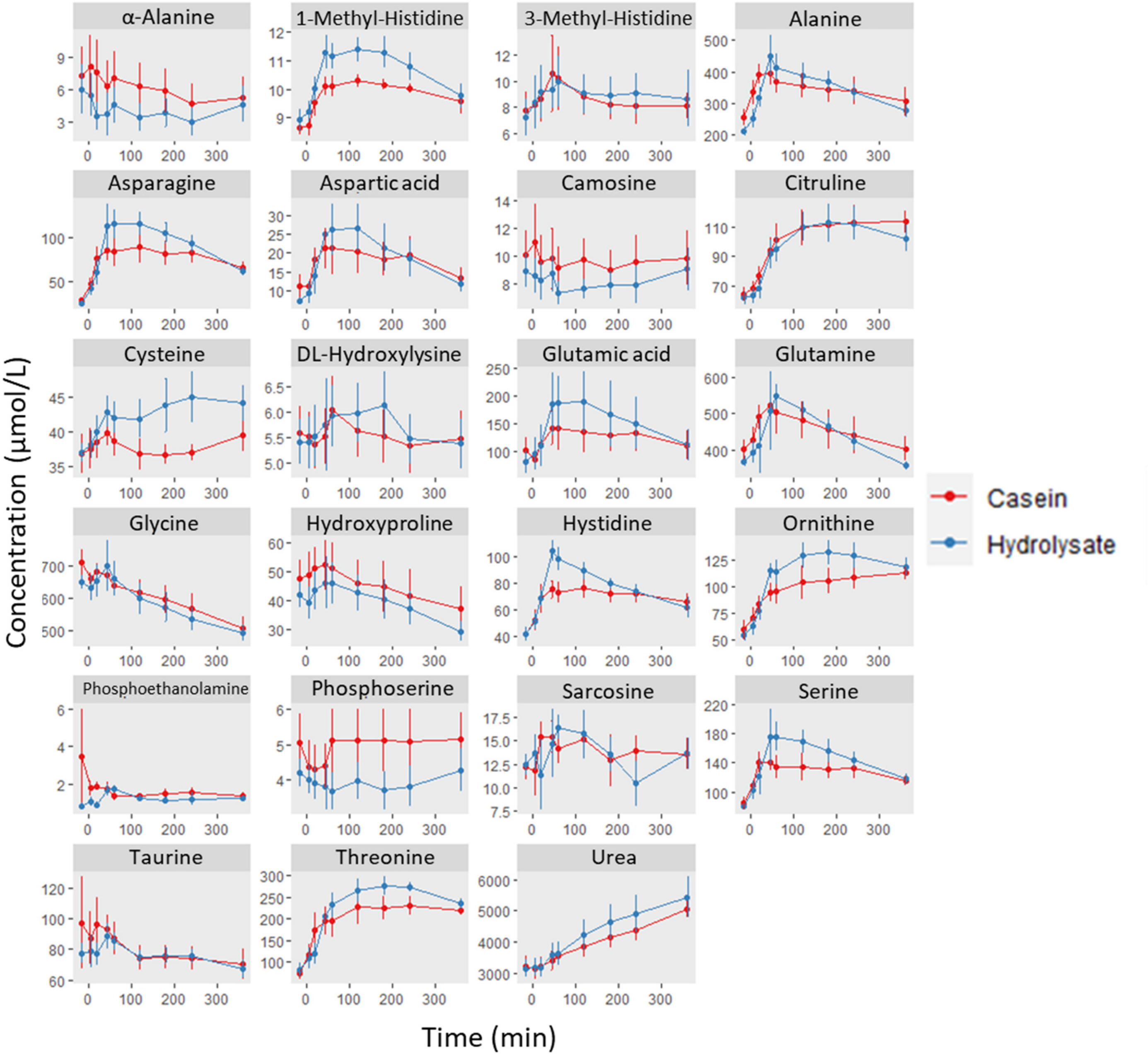

Figure S6. Concentration of amino acids in plasma of pigs (*n* =6) at baseline (-15 min) and 5, 20, 45, 60, 120, 180, 240 and 360 min after intake of casein or hydrolysate.

Table S1. Pig endogenous proteins identified in duodenal effluents.

Protein	Accession number[1]	Function
Pancreatic triacylglycerol lipase	P00591	Digestive enzymes
Lipoprotein lipase	P49923	
Pepsin A	P00791	
Chymotrypsin-like elastase family member 2A	P08419	
Pancreatic alpha-amylase		
Trypsin	P00761	
Dipeptidyl peptidase 4	P22411	
Carboxypeptidase A1	P09954	
Aminopeptidase	P15145	
Pro-glucagon	P01274	Enteroendocrine regulation
Appetite-regulating hormone (Ghrelin)	Q9GKY5	
Gastrin-releasing peptide	P63153	
Gastrin/cholecystokinin type B receptor	A0A287A2K5	
Mucin-5AC	A0A4X1UGK3	Lubrication, maintenance of integrity of tissue lining
Serum albumin	P08835	Maintenance of colloid osmotic pressure (from plasma into stomach and intestine)

[1] Compiled from the UniProtKB Protein Database
