# Supplementary figures and images for: Peptidomic Characterization and Amino Acid Availability after Intake of Casein vs. a Casein Hydrolysate in a Pig Model
Source: Nutrients. 2023 Feb 21;15(5):1065. doi: 10.3390/nu15051065 (PMC10005447; doi:10.3390/nu15051065)

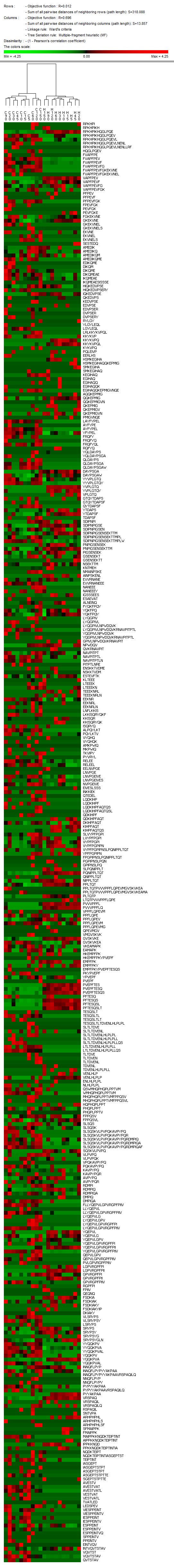

Supplement: Supplementary file 1 [file nutrients-15-01065-s001.zip › nutrients-2209596-Full PermutMatrix results.png]
